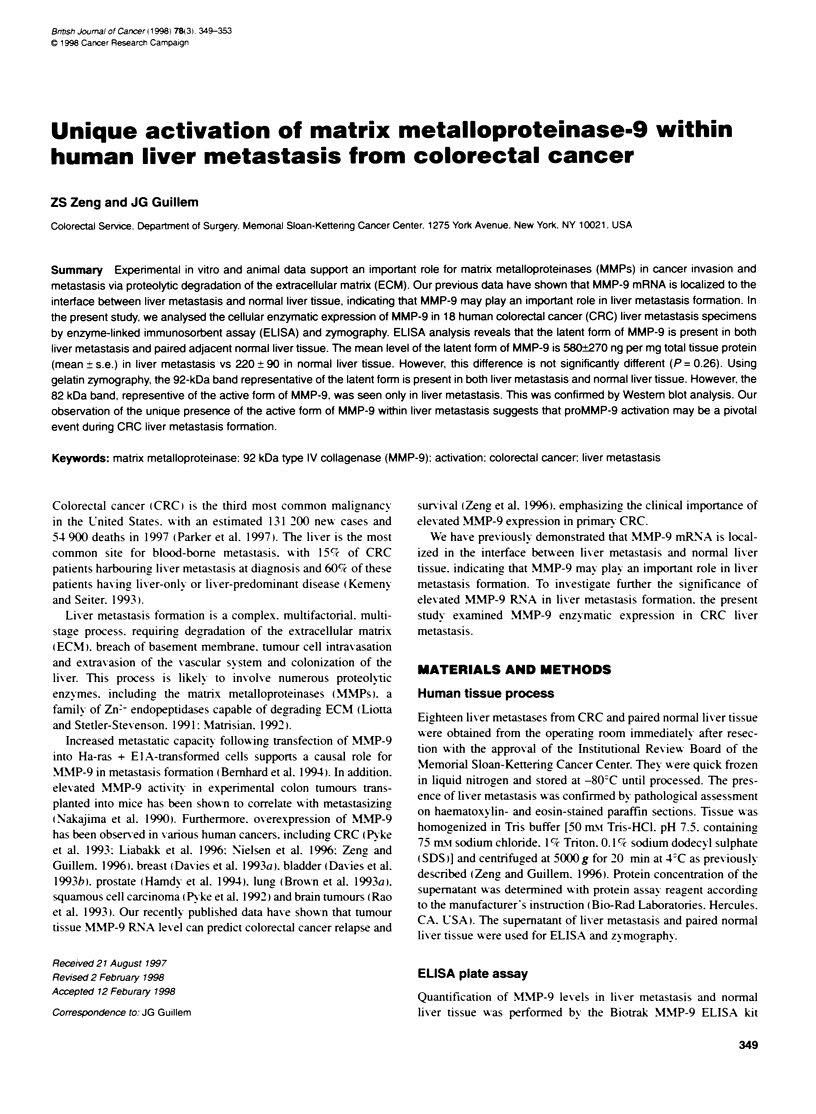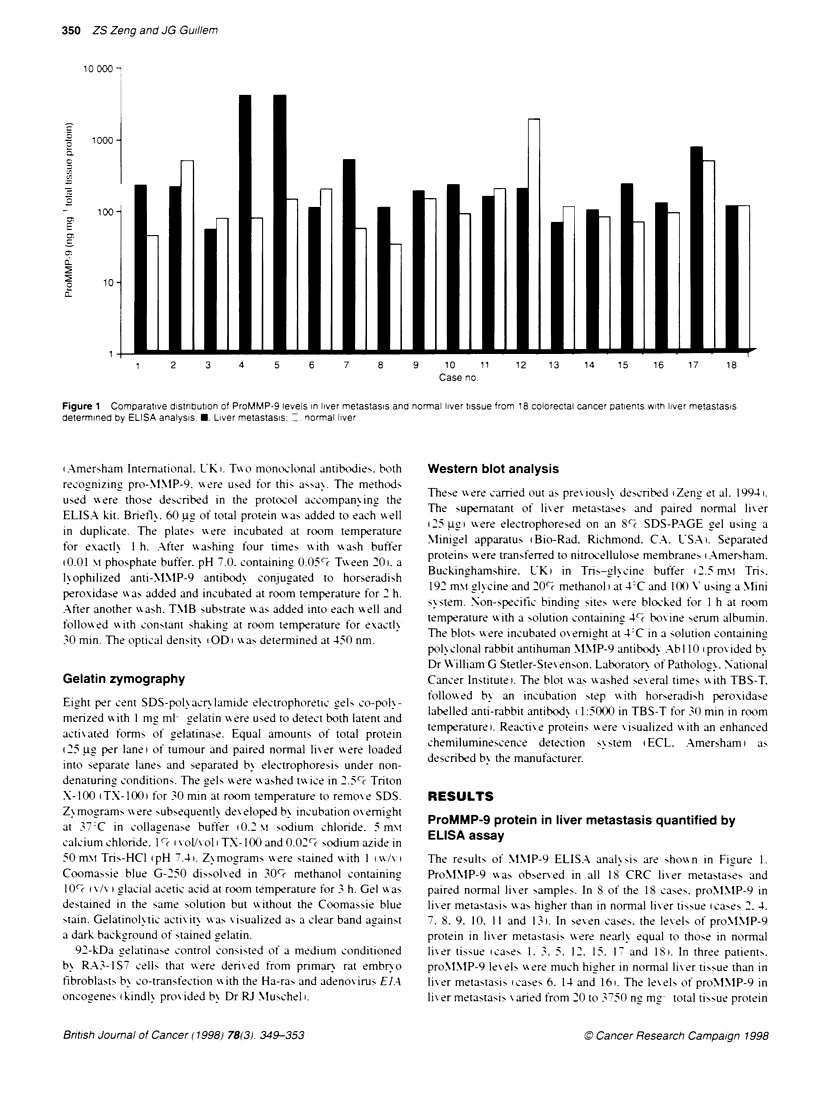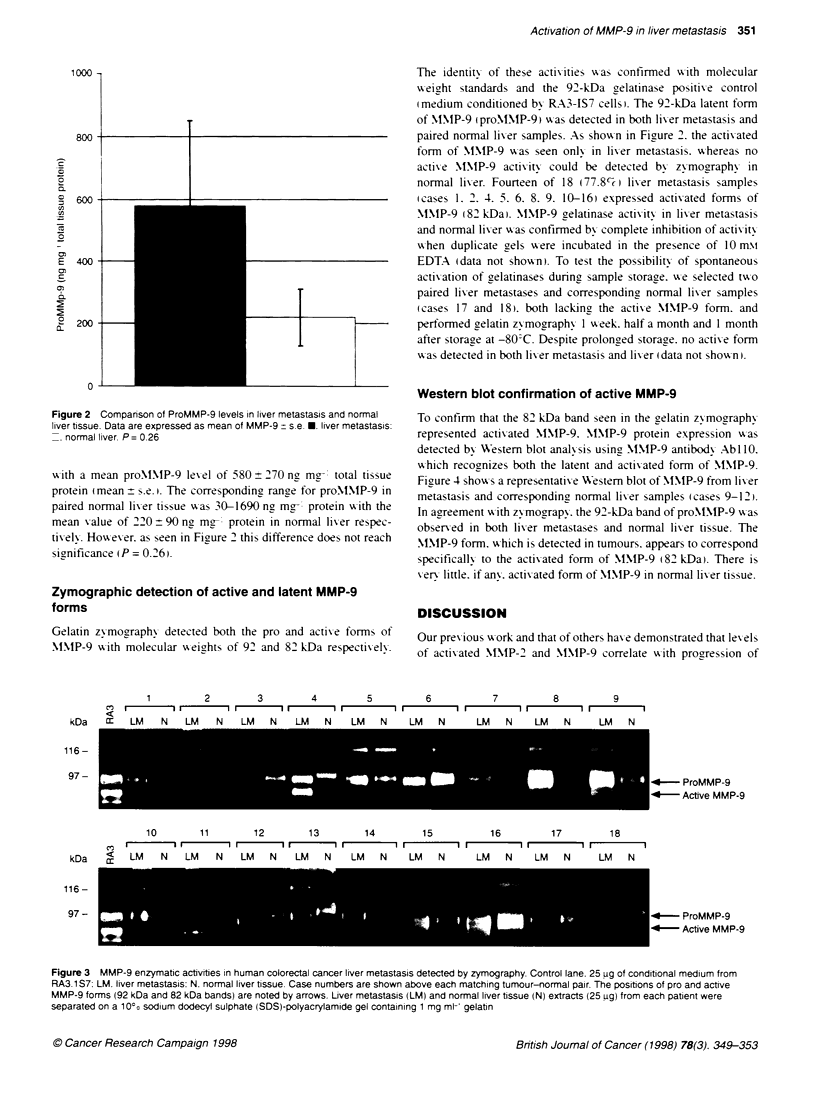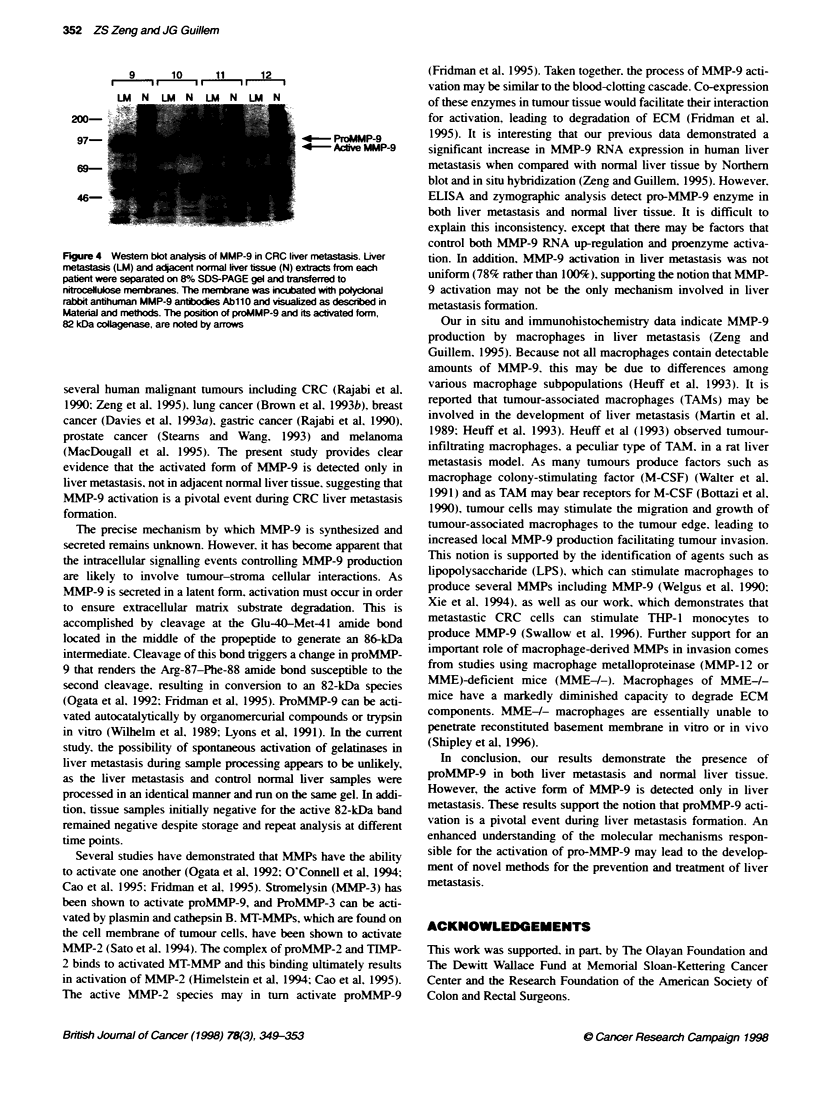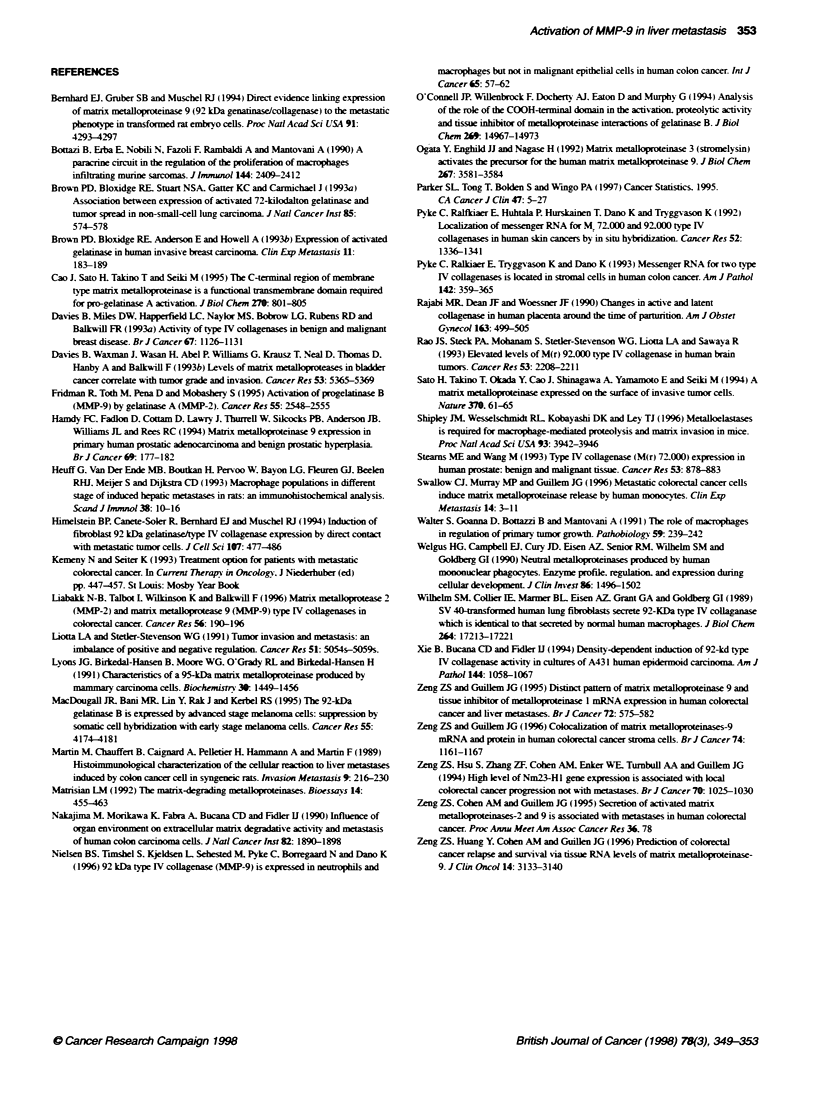# Unique activation of matrix metalloproteinase-9 within human liver metastasis from colorectal cancer.

**DOI:** 10.1038/bjc.1998.497

**Published:** 1998-08

**Authors:** Z. S. Zeng, J. G. Guillem

**Affiliations:** Colorectal Service, Department of Surgery, Memorial Sloan-Kettering Cancer Center, New York, NY 10021, USA.

## Abstract

**Images:**


British Journal of Cancer (1998) 78(3), 349–353
© 1998 Cancer Research Campaign

### Unique activation of matrix metalloproteinase-9 within human liver metastasis from colorectal cancer

**ZS Zeng and JG Guillem**

Colorectal Service, Department of Surgery, Memorial Sloan-Kettering Cancer Center, 1275 York Avenue, New York, NY 10021, USA

**Summary** Experimental in vitro and animal data support an important role for matrix metalloproteinases (MMPs) in cancer invasion and metastasis via proteolytic degradation of the extracellular matrix (ECM). Our previous data have shown that MMP-9 mRNA is localized to the interface between liver metastasis and normal liver tissue, indicating that MMP-9 may play an important role in liver metastasis formation. In the present study, we analysed the cellular enzymatic expression of MMP-9 in 18 human colorectal cancer (CRC) liver metastasis specimens by enzyme-linked immunosorbent assay (ELISA) and zymography. ELISA analysis reveals that the latent form of MMP-9 is present in both liver metastasis and paired adjacent normal liver tissue. The mean level of the latent form of MMP-9 is 580±270 ng per mg total tissue protein (mean ± s.e.) in liver metastasis vs 220 ± 90 in normal liver tissue. However, this difference is not significantly different ($P = 0.26$). Using gelatin zymography, the 92-kDa band representative of the latent form is present in both liver metastasis and normal liver tissue. However, the 82 kDa band, representive of the active form of MMP-9, was seen only in liver metastasis. This was confirmed by Western blot analysis. Our observation of the unique presence of the active form of MMP-9 within liver metastasis suggests that proMMP-9 activation may be a pivotal event during CRC liver metastasis formation.

**Keywords:** matrix metalloproteinase; 92 kDa type IV collagenase (MMP-9); activation; colorectal cancer; liver metastasis

Colorectal cancer (CRC) is the third most common malignancy in the United States, with an estimated 131 200 new cases and 54 900 deaths in 1997 (Parker et al, 1997). The liver is the most common site for blood-borne metastasis, with 15% of CRC patients harbouring liver metastasis at diagnosis and 60% of these patients having liver-only or liver-predominant disease (Kemeny and Seiter, 1993).

Liver metastasis formation is a complex, multifactorial, multistage process, requiring degradation of the extracellular matrix (ECM), breach of basement membrane, tumour cell intravasation and extravasion of the vascular system and colonization of the liver. This process is likely to involve numerous proteolytic enzymes, including the matrix metalloproteinases (MMPs), a family of $Zn^{2+}$ endopeptidases capable of degrading ECM (Liotta and Stetler-Stevenson, 1991; Matrisian, 1992).

Increased metastatic capacity following transfection of MMP-9 into Ha-ras + E1A-transformed cells supports a causal role for MMP-9 in metastasis formation (Bernhard et al, 1994). In addition, elevated MMP-9 activity in experimental colon tumours transplanted into mice has been shown to correlate with metastasizing (Nakajima et al, 1990). Furthermore, overexpression of MMP-9 has been observed in various human cancers, including CRC (Pyke et al, 1993; Liabakk et al, 1996; Nielsen et al, 1996; Zeng and Guillem, 1996), breast (Davies et al, 1993*a*), bladder (Davies et al, 1993*b*), prostate (Hamdy et al, 1994), lung (Brown et al, 1993*a*), squamous cell carcinoma (Pyke et al, 1992) and brain tumours (Rao et al, 1993). Our recently published data have shown that tumour tissue MMP-9 RNA level can predict colorectal cancer relapse and survival (Zeng et al, 1996), emphasizing the clinical importance of elevated MMP-9 expression in primary CRC.

We have previously demonstrated that MMP-9 mRNA is localized in the interface between liver metastasis and normal liver tissue, indicating that MMP-9 may play an important role in liver metastasis formation. To investigate further the significance of elevated MMP-9 RNA in liver metastasis formation, the present study examined MMP-9 enzymatic expression in CRC liver metastasis.

### MATERIALS AND METHODS

### Human tissue process

Eighteen liver metastases from CRC and paired normal liver tissue were obtained from the operating room immediately after resection with the approval of the Institutional Review Board of the Memorial Sloan-Kettering Cancer Center. They were quick frozen in liquid nitrogen and stored at –80°C until processed. The presence of liver metastasis was confirmed by pathological assessment on haematoxylin- and eosin-stained paraffin sections. Tissue was homogenized in Tris buffer [50 mM Tris-HCl, pH 7.5, containing 75 mM sodium chloride, 1% Triton, 0.1% sodium dodecyl sulphate (SDS)] and centrifuged at 5000 *g* for 20 min at 4°C as previously described (Zeng and Guillem, 1996). Protein concentration of the supernatant was determined with protein assay reagent according to the manufacturer's instruction (Bio-Rad Laboratories, Hercules, CA, USA). The supernatant of liver metastasis and paired normal liver tissue were used for ELISA and zymography.

### ELISA plate assay

Quantification of MMP-9 levels in liver metastasis and normal liver tissue was performed by the Biotrak MMP-9 ELISA kit

*Received 21 August 1997*
*Revised 2 February 1998*
*Accepted 12 Feburary 1998*

*Correspondence to:* JG Guillem

**Figure 1** Comparative distribution of ProMMP-9 levels in liver metastasis and normal liver tissue from 18 colorectal cancer patients with liver metastasis determined by ELISA analysis. ■, Liver metastasis; □, normal liver

(Amersham International, UK). Two monoclonal antibodies, both recognizing pro-MMP-9, were used for this assay. The methods used were those described in the protocol accompanying the ELISA kit. Briefly, 60 µg of total protein was added to each well in duplicate. The plates were incubated at room temperature for exactly 1 h. After washing four times with wash buffer (0.01 M phosphate buffer, pH 7.0, containing 0.05% Tween 20), a lyophilized anti-MMP-9 antibody conjugated to horseradish peroxidase was added and incubated at room temperature for 2 h. After another wash, TMB substrate was added into each well and followed with constant shaking at room temperature for exactly 30 min. The optical density (OD) was determined at 450 nm.

### Gelatin zymography

Eight per cent SDS-polyacrylamide electrophoretic gels co-polymerized with 1 mg $ml^{-1}$ gelatin were used to detect both latent and activated forms of gelatinase. Equal amounts of total protein (25 µg per lane) of tumour and paired normal liver were loaded into separate lanes and separated by electrophoresis under non-denaturing conditions. The gels were washed twice in 2.5% Triton X-100 (TX-100) for 30 min at room temperature to remove SDS. Zymograms were subsequently developed by incubation overnight at 37°C in collagenase buffer (0.2 M sodium chloride, 5 mM calcium chloride, 1% (vol/vol) TX-100 and 0.02% sodium azide in 50 mM Tris-HCl (pH 7.4). Zymograms were stained with 1 (w/v) Coomassie blue G-250 dissolved in 30% methanol containing 10% (v/v) glacial acetic acid at room temperature for 3 h. Gel was destained in the same solution but without the Coomassie blue stain. Gelatinolytic activity was visualized as a clear band against a dark background of stained gelatin.

92-kDa gelatinase control consisted of a medium conditioned by RA3-1S7 cells that were derived from primary rat embryo fibroblasts by co-transfection with the Ha-ras and adenovirus *E1A* oncogenes (kindly provided by Dr RJ Muschel).

### Western blot analysis

These were carried out as previously described (Zeng et al, 1994). The supernatant of liver metastases and paired normal liver (25 µg) were electrophoresed on an 8% SDS-PAGE gel using a Minigel apparatus (Bio-Rad, Richmond, CA, USA). Separated proteins were transferred to nitrocellulose membranes (Amersham, Buckinghamshire, UK) in Tris–glycine buffer (2.5 mM Tris, 192 mM glycine and 20% methanol) at 4°C and 100 V using a Mini system. Non-specific binding sites were blocked for 1 h at room temperature with a solution containing 4% bovine serum albumin. The blots were incubated overnight at 4°C in a solution containing polyclonal rabbit antihuman MMP-9 antibody Ab110 (provided by Dr William G Stetler-Stevenson, Laboratory of Pathology, National Cancer Institute). The blot was washed several times with TBS-T, followed by an incubation step with horseradish peroxidase labelled anti-rabbit antibody (1:5000 in TBS-T for 30 min in room temperature). Reactive proteins were visualized with an enhanced chemiluminescence detection system (ECL, Amersham) as described by the manufacturer.

### RESULTS

### ProMMP-9 protein in liver metastasis quantified by ELISA assay

The results of MMP-9 ELISA analysis are shown in Figure 1. ProMMP-9 was observed in all 18 CRC liver metastases and paired normal liver samples. In 8 of the 18 cases, proMMP-9 in liver metastasis was higher than in normal liver tissue (cases 2, 4, 7, 8, 9, 10, 11 and 13). In seven cases, the levels of proMMP-9 protein in liver metastasis were nearly equal to those in normal liver tissue (cases 1, 3, 5, 12, 15, 17 and 18). In three patients, proMMP-9 levels were much higher in normal liver tissue than in liver metastasis (cases 6, 14 and 16). The levels of proMMP-9 in liver metastasis varied from 20 to 3750 ng $mg^{-1}$ total tissue protein

© Cancer Research Campaign 1998

Figure 2 Comparison of ProMMP-9 levels in liver metastasis and normal liver tissue. Data are expressed as mean of MMP-9 ± s.e. ■, liver metastasis; □, normal liver. $P = 0.26$

with a mean proMMP-9 level of $580 \pm 270$ ng mg⁻¹ total tissue protein (mean ± s.e.). The corresponding range for proMMP-9 in paired normal liver tissue was 30–1690 ng mg⁻¹ protein with the mean value of $220 \pm 90$ ng mg⁻¹ protein in normal liver respectively. However, as seen in Figure 2 this difference does not reach significance ($P = 0.26$).

### Zymographic detection of active and latent MMP-9 forms

Gelatin zymography detected both the pro and active forms of MMP-9 with molecular weights of 92 and 82 kDa respectively. The identity of these activities was confirmed with molecular weight standards and the 92-kDa gelatinase positive control (medium conditioned by RA3-IS7 cells). The 92-kDa latent form of MMP-9 (proMMP-9) was detected in both liver metastasis and paired normal liver samples. As shown in Figure 2, the activated form of MMP-9 was seen only in liver metastasis, whereas no active MMP-9 activity could be detected by zymography in normal liver. Fourteen of 18 (77.8%) liver metastasis samples (cases 1, 2, 4, 5, 6, 8, 9, 10–16) expressed activated forms of MMP-9 (82 kDa). MMP-9 gelatinase activity in liver metastasis and normal liver was confirmed by complete inhibition of activity when duplicate gels were incubated in the presence of 10 mM EDTA (data not shown). To test the possibility of spontaneous activation of gelatinases during sample storage, we selected two paired liver metastases and corresponding normal liver samples (cases 17 and 18), both lacking the active MMP-9 form, and performed gelatin zymography 1 week, half a month and 1 month after storage at –80°C. Despite prolonged storage, no active form was detected in both liver metastasis and liver (data not shown).

### Western blot confirmation of active MMP-9

To confirm that the 82 kDa band seen in the gelatin zymography represented activated MMP-9, MMP-9 protein expression was detected by Western blot analysis using MMP-9 antibody Ab110, which recognizes both the latent and activated form of MMP-9. Figure 4 shows a representative Western blot of MMP-9 from liver metastasis and corresponding normal liver samples (cases 9–12). In agreement with zymograpy, the 92-kDa band of proMMP-9 was observed in both liver metastases and normal liver tissue. The MMP-9 form, which is detected in tumours, appears to correspond specifically to the activated form of MMP-9 (82 kDa). There is very little, if any, activated form of MMP-9 in normal liver tissue.

### DISCUSSION

Our previous work and that of others have demonstrated that levels of activated MMP-2 and MMP-9 correlate with progression of

Figure 3 MMP-9 enzymatic activities in human colorectal cancer liver metastasis detected by zymography. Control lane, 25 µg of conditional medium from RA3.1S7; LM, liver metastasis; N, normal liver tissue. Case numbers are shown above each matching tumour–normal pair. The positions of pro and active MMP-9 forms (92 kDa and 82 kDa bands) are noted by arrows. Liver metastasis (LM) and normal liver tissue (N) extracts (25 µg) from each patient were separated on a 10% sodium dodecyl sulphate (SDS)-polyacrylamide gel containing 1 mg ml⁻¹ gelatin

© Cancer Research Campaign 1998

**Figure 4** Western blot analysis of MMP-9 in CRC liver metastasis. Liver metastasis (LM) and adjacent normal liver tissue (N) extracts from each patient were separated on 8% SDS-PAGE gel and transferred to nitrocellulose membranes. The membrane was incubated with polyclonal rabbit antihuman MMP-9 antibodies Ab110 and visualized as described in Material and methods. The position of proMMP-9 and its activated form, 82 kDa collagenase, are noted by arrows several human malignant tumours including CRC (Rajabi et al, 1990; Zeng et al, 1995), lung cancer (Brown et al, 1993*b*), breast cancer (Davies et al, 1993*a*), gastric cancer (Rajabi et al, 1990), prostate cancer (Stearns and Wang, 1993) and melanoma (MacDougall et al, 1995). The present study provides clear evidence that the activated form of MMP-9 is detected only in liver metastasis, not in adjacent normal liver tissue, suggesting that MMP-9 activation is a pivotal event during CRC liver metastasis formation.

The precise mechanism by which MMP-9 is synthesized and secreted remains unknown. However, it has become apparent that the intracellular signalling events controlling MMP-9 production are likely to involve tumour–stroma cellular interactions. As MMP-9 is secreted in a latent form, activation must occur in order to ensure extracellular matrix substrate degradation. This is accomplished by cleavage at the Glu-40–Met-41 amide bond located in the middle of the propeptide to generate an 86-kDa intermediate. Cleavage of this bond triggers a change in proMMP-9 that renders the Arg-87–Phe-88 amide bond susceptible to the second cleavage, resulting in conversion to an 82-kDa species (Ogata et al, 1992; Fridman et al, 1995). ProMMP-9 can be activated autocatalytically by organomercurial compounds or trypsin in vitro (Wilhelm et al, 1989; Lyons et al, 1991). In the current study, the possibility of spontaneous activation of gelatinases in liver metastasis during sample processing appears to be unlikely, as the liver metastasis and control normal liver samples were processed in an identical manner and run on the same gel. In addition, tissue samples initially negative for the active 82-kDa band remained negative despite storage and repeat analysis at different time points.

Several studies have demonstrated that MMPs have the ability to activate one another (Ogata et al, 1992; O'Connell et al, 1994; Cao et al, 1995; Fridman et al, 1995). Stromelysin (MMP-3) has been shown to activate proMMP-9, and ProMMP-3 can be activated by plasmin and cathepsin B. MT-MMPs, which are found on the cell membrane of tumour cells, have been shown to activate MMP-2 (Sato et al, 1994). The complex of proMMP-2 and TIMP-2 binds to activated MT-MMP and this binding ultimately results in activation of MMP-2 (Himelstein et al, 1994; Cao et al, 1995). The active MMP-2 species may in turn activate proMMP-9 (Fridman et al, 1995). Taken together, the process of MMP-9 activation may be similar to the blood-clotting cascade. Co-expression of these enzymes in tumour tissue would facilitate their interaction for activation, leading to degradation of ECM (Fridman et al, 1995). It is interesting that our previous data demonstrated a significant increase in MMP-9 RNA expression in human liver metastasis when compared with normal liver tissue by Northern blot and in situ hybridization (Zeng and Guillem, 1995). However, ELISA and zymographic analysis detect pro-MMP-9 enzyme in both liver metastasis and normal liver tissue. It is difficult to explain this inconsistency, except that there may be factors that control both MMP-9 RNA up-regulation and proenzyme activation. In addition, MMP-9 activation in liver metastasis was not uniform (78% rather than 100%), supporting the notion that MMP-9 activation may not be the only mechanism involved in liver metastasis formation.

Our in situ and immunohistochemistry data indicate MMP-9 production by macrophages in liver metastasis (Zeng and Guillem, 1995). Because not all macrophages contain detectable amounts of MMP-9, this may be due to differences among various macrophage subpopulations (Heuff et al, 1993). It is reported that tumour-associated macrophages (TAMs) may be involved in the development of liver metastasis (Martin et al, 1989; Heuff et al, 1993). Heuff et al (1993) observed tumour-infiltrating macrophages, a peculiar type of TAM, in a rat liver metastasis model. As many tumours produce factors such as macrophage colony-stimulating factor (M-CSF) (Walter et al, 1991) and as TAM may bear receptors for M-CSF (Bottazi et al, 1990), tumour cells may stimulate the migration and growth of tumour-associated macrophages to the tumour edge, leading to increased local MMP-9 production facilitating tumour invasion. This notion is supported by the identification of agents such as lipopolysaccharide (LPS), which can stimulate macrophages to produce several MMPs including MMP-9 (Welgus et al, 1990; Xie et al, 1994), as well as our work, which demonstrates that metastastic CRC cells can stimulate THP-1 monocytes to produce MMP-9 (Swallow et al, 1996). Further support for an important role of macrophage-derived MMPs in invasion comes from studies using macrophage metalloproteinase (MMP-12 or MME)-deficient mice (MME-/-). Macrophages of MME-/- mice have a markedly diminished capacity to degrade ECM components. MME-/- macrophages are essentially unable to penetrate reconstituted basement membrane in vitro or in vivo (Shipley et al, 1996).

In conclusion, our results demonstrate the presence of proMMP-9 in both liver metastasis and normal liver tissue. However, the active form of MMP-9 is detected only in liver metastasis. These results support the notion that proMMP-9 activation is a pivotal event during liver metastasis formation. An enhanced understanding of the molecular mechanisms responsible for the activation of pro-MMP-9 may lead to the development of novel methods for the prevention and treatment of liver metastasis.

### ACKNOWLEDGEMENTS

This work was supported, in part, by The Olayan Foundation and The Dewitt Wallace Fund at Memorial Sloan-Kettering Cancer Center and the Research Foundation of the American Society of Colon and Rectal Surgeons.

© Cancer Research Campaign 1998

### REFERENCES

Bernhard EJ. Gruber SB and Muschel RJ (1994) Direct evidence linking expression of matrix metalloproteinase 9 (92 kDa genatinase/collagenase) to the metastatic phenotype in transformed rat embryo cells. *Proc Natl Acad Sci USA* **91**: 4293–4297

Bottazi B. Erba E. Nobili N. Fazoli F. Rambaldi A and Mantovani A (1990) A paracrine circuit in the regulation of the proliferation of macrophages infiltrating murine sarcomas. *J Immunol* **144**: 2409–2412

Brown PD. Bloxidge RE. Stuart NSA. Gatter KC and Carmichael J (1993*a*) Association between expression of activated 72-kilodalton gelatinase and tumor spread in non-small-cell lung carcinoma. *J Natl Cancer Inst* **85**: 574–578

Brown PD. Bloxidge RE. Anderson E and Howell A (1993*b*) Expression of activated gelatinase in human invasive breast carcinoma. *Clin Exp Metastasis* **11**: 183–189

Cao J. Sato H. Takino T and Seiki M (1995) The C-terminal region of membrane type matrix metalloproteinase is a functional transmembrane domain required for pro-gelatinase A activation. *J Biol Chem* **270**: 801–805

Davies B. Miles DW. Happerfield LC. Naylor MS. Bobrow LG. Rubens RD and Balkwill FR (1993*a*) Activity of type IV collagenases in benign and malignant breast disease. *Br J Cancer* **67**: 1126–1131

Davies B. Waxman J. Wasan H. Abel P. Williams G. Krausz T. Neal D. Thomas D. Hanby A and Balkwill F (1993*b*) Levels of matrix metalloproteases in bladder cancer correlate with tumor grade and invasion. *Cancer Res* **53**: 5365–5369

Fridman R. Toth M. Pena D and Mobashery S (1995) Activation of progelatinase B (MMP-9) by gelatinase A (MMP-2). *Cancer Res* **55**: 2548–2555

Hamdy FC. Fadlon D. Cottam D. Lawry J. Thurrell W. Silcocks PB. Anderson JB. Williams JL and Rees RC (1994) Matrix metalloproteinase 9 expression in primary human prostatic adenocarcinoma and benign prostatic hyperplasia. *Br J Cancer* **69**: 177–182

Heuff G. Van Der Ende MB. Boutkan H. Pervoo W. Bayon LG. Fleuren GJ. Beelen RHJ. Meijer S and Dijkstra CD (1993) Macrophage populations in different stage of induced hepatic metastases in rats: an immunohistochemical analysis. *Scand J Immnol* **38**: 10–16

Himelstein BP. Canete-Soler R. Bernhard EJ and Muschel RJ (1994) Induction of fibroblast 92 kDa gelatinase/type IV collagenase expression by direct contact with metastatic tumor cells. *J Cell Sci* **107**: 477–486

Kemeny N and Seiter K (1993) Treatment option for patients with metastatic colorectal cancer. In *Current Therapy in Oncology*. J Niederhuber (ed) pp. 447–457. St Louis: Mosby Year Book

Liabakk N-B. Talbot I. Wilkinson K and Balkwill F (1996) Matrix metalloprotease 2 (MMP-2) and matrix metalloprotease 9 (MMP-9) type IV collagenases in colorectal cancer. *Cancer Res* **56**: 190–196

Liotta LA and Stetler-Stevenson WG (1991) Tumor invasion and metastasis: an imbalance of positive and negative regulation. *Cancer Res* **51**: 5054s–5059s.

Lyons JG. Birkedal-Hansen B. Moore WG. O'Grady RL and Birkedal-Hansen H (1991) Characteristics of a 95-kDa matrix metalloproteinase produced by mammary carcinoma cells. *Biochemistry* **30**: 1449–1456

MacDougall JR. Bani MR. Lin Y. Rak J and Kerbel RS (1995) The 92-kDa gelatinase B is expressed by advanced stage melanoma cells: suppression by somatic cell hybridization with early stage melanoma cells. *Cancer Res* **55**: 4174–4181

Martin M. Chauffert B. Caignard A. Pelletier H. Hammann A and Martin F (1989) Histoimmunological characterization of the cellular reaction to liver metastases induced by colon cancer cell in syngeneic rats. *Invasion Metastasis* **9**: 216–230

Matrisian LM (1992) The matrix-degrading metalloproteinases. *Bioessays* **14**: 455–463

Nakajima M. Morikawa K. Fabra A. Bucana CD and Fidler IJ (1990) Influence of organ environment on extracellular matrix degradative activity and metastasis of human colon carcinoma cells. *J Natl Cancer Inst* **82**: 1890–1898

Nielsen BS. Timshel S. Kjeldsen L. Sehested M. Pyke C. Borregaard N and Dano K (1996) 92 kDa type IV collagenase (MMP-9) is expressed in neutrophils and macrophages but not in malignant epithelial cells in human colon cancer. *Int J Cancer* **65**: 57–62

O'Connell JP. Willenbrock F. Docherty AJ. Eaton D and Murphy G (1994) Analysis of the role of the COOH-terminal domain in the activation. proteolytic activity and tissue inhibitor of metalloproteinase interactions of gelatinase B. *J Biol Chem* **269**: 14967–14973

Ogata Y. Enghild JJ and Nagase H (1992) Matrix metalloproteinase 3 (stromelysin) activates the precursor for the human matrix metalloproteinase 9. *J Biol Chem* **267**: 3581–3584

Parker SL. Tong T. Bolden S and Wingo PA (1997) Cancer Statistics. 1995. *CA Cancer J Clin* **47**: 5–27

Pyke C. Ralfkiaer E. Huhtala P. Hurskainen T. Dano K and Tryggvason K (1992) Localization of messenger RNA for $M_r$ 72.000 and 92.000 type IV collagenases in human skin cancers by in situ hybridization. *Cancer Res* **52**: 1336–1341

Pyke C. Ralkiaer E. Tryggvason K and Dano K (1993) Messenger RNA for two type IV collagenases is located in stromal cells in human colon cancer. *Am J Pathol* **142**: 359–365

Rajabi MR. Dean JF and Woessner JF (1990) Changes in active and latent collagenase in human placenta around the time of parturition. *Am J Obstet Gynecol* **163**: 499–505

Rao JS. Steck PA. Mohanam S. Stetler-Stevenson WG. Liotta LA and Sawaya R (1993) Elevated levels of M(r) 92.000 type IV collagenase in human brain tumors. *Cancer Res* **53**: 2208–2211

Sato H. Takino T. Okada Y. Cao J. Shinagawa A. Yamamoto E and Seiki M (1994) A matrix metalloproteinase expressed on the surface of invasive tumor cells. *Nature* **370**. 61–65

Shipley JM. Wesselschmidt RL. Kobayashi DK and Ley TJ (1996) Metalloelastases is required for macrophage-mediated proteolysis and matrix invasion in mice. *Proc Natl Acad Sci USA* **93**: 3942–3946

Stearns ME and Wang M (1993) Type IV collagenase (M(r) 72.000) expression in human prostate: benign and malignant tissue. *Cancer Res* **53**: 878–883

Swallow CJ. Murray MP and Guillem JG (1996) Metastatic colorectal cancer cells induce matrix metalloproteinase release by human monocytes. *Clin Exp Metastasis* **14**: 3–11

Walter S. Goanna D. Bottazzi B and Mantovani A (1991) The role of macrophages in regulation of primary tumor growth. *Pathobiology* **59**: 239–242

Welgus HG. Campbell EJ. Cury JD. Eisen AZ. Senior RM. Wilhelm SM and Goldberg GI (1990) Neutral metalloproteinases produced by human mononuclear phagocytes. Enzyme profile. regulation. and expression during cellular development. *J Clin Invest* **86**: 1496–1502

Wilhelm SM. Collier IE. Marmer BL. Eisen AZ. Grant GA and Goldberg GI (1989) SV 40-transformed human lung fibroblasts secrete 92-KDa type IV collaganase which is identical to that secreted by normal human macrophages. *J Biol Chem* **264**: 17213–17221

Xie B. Bucana CD and Fidler IJ (1994) Density-dependent induction of 92-kd type IV collagenase activity in cultures of A431 human epidermoid carcinoma. *Am J Pathol* **144**: 1058–1067

Zeng ZS and Guillem JG (1995) Distinct pattern of matrix metalloproteinase 9 and tissue inhibitor of metalloproteinase 1 mRNA expression in human colorectal cancer and liver metastases. *Br J Cancer* **72**: 575–582

Zeng ZS and Guillem JG (1996) Colocalization of matrix metalloproteinases-9 mRNA and protein in human colorectal cancer stroma cells. *Br J Cancer* **74**: 1161–1167

Zeng ZS. Hsu S. Zhang ZF. Cohen AM. Enker WE. Turnbull AA and Guillem JG (1994) High level of Nm23-H1 gene expression is associated with local colorectal cancer progression not with metastases. *Br J Cancer* **70**: 1025–1030

Zeng ZS. Cohen AM and Guillem JG (1995) Secretion of activated matrix metalloproteinases-2 and 9 is associated with metastases in human colorectal cancer. *Proc Annu Meet Am Assoc Cancer Res* **36**. 78

Zeng ZS. Huang Y. Cohen AM and Guillen JG (1996) Prediction of colorectal cancer relapse and survival via tissue RNA levels of matrix metalloproteinase-9. *J Clin Oncol* **14**: 3133–3140

© Cancer Research Campaign 1998